# Evaluation of Fluoride and Selected Chemical Parameters in Kombucha Derived from White, Green, Black and Red Tea

**DOI:** 10.1007/s12011-020-02445-9

**Published:** 2020-11-08

**Authors:** Karolina Jakubczyk, Izabela Gutowska, Justyna Antoniewicz, Katarzyna Janda

**Affiliations:** 1 Department of Human Nutrition and Metabolomics, Pomeranian Medical University in Szczecin, 24 Broniewskiego Street, 71-460 Szczecin, Poland; 2 Department of Medical Chemistry, Pomeranian Medical University in Szczecin, 72 Powstańców Wlkp. Street, 70-111 Szczecin, Poland

**Keywords:** Kombucha, Fermented tea, Toxicity, Safety, Beverage, Acetic acid, Fluoride

## Abstract

Kombucha dates back thousands of years and is reported to have originated in East Asia. It is produced by fermenting tea with added sugar using SCOBY (symbiotic culture of bacteria and yeast). Its health benefits can be attributed to the metabolites produced during the fermentation process. Valuable ingredients of this fermented tea beverage include acetic acid, glucuronic acid, vitamins, enzymes, sugars and polyphenols. Tea, and consequently kombucha, contains numerous minerals, and one of them is fluoride. Under physiological conditions, fluoride plays a significant role in hard tissue mineralisation processes. However, even at low concentrations with long-term exposure, fluorides may accumulate in the body and cause a range of detrimental effects. Kombucha is traditionally brewed with black tea, but these days it is becoming increasingly popular to use other types of tea to make it, which may significantly affect its composition and health-promoting effects. The aim of the study was to evaluate the fluoride content in kombucha beverages derived from black, green, white and red tea. Fluoride content was measured at different time points during fermentation. The potentiometric method was used to determine the content of fluoride ions. It was demonstrated that kombucha is a major dietary source of fluoride (0.42–0.93 mg/L) and that the type of tea used has a significant effect on its chemical composition. Therefore, it recommended to make the beverage with white or red tea, due to the lowest fluoride content and ensure food safety.

Graphical Abstract
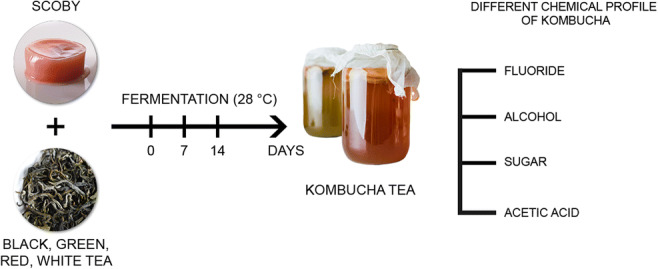

## Introduction

Kombucha is a fermented tea beverage made using a symbiotic culture of bacteria and yeast (SCOBY), commonly called a “mother” or “mushroom”. Kombucha is made from tea by adding sugar (10%), starter tea from a previous batch (10%) and the mother culture. The SCOBY added to sweetened tea initiates fermentation, which leads to the production of numerous bioactive components. The tea is left at room temperature to ferment for 7–21 days. Kombucha can be made with different types of tea, including green tea and fermented (oxidised) varieties, like red, black and yellow tea [1–3]. Still, black tea and white sugar (sucrose) are regarded as the traditional and at the same time the best ingredients, making for the optimal composition of the finished beverage and its health benefits. The taste of kombucha is described as sour and fruity, slightly carbonated, becoming similar to the taste of wine vinegar after a few days’ storage [2]. Studies demonstrated the antimicrobial, antioxidant and antidiabetic properties of kombucha, lowering cholesterol and boosting the immune system, as well as stimulating liver detoxification [4]. Kombucha beverages were also found to contain minerals, mainly originating from the tea substrate (F, K, Mn), vitamins (E, K, B) and amino acids (notably theanine, a derivative of glutamine), as well as other compounds produced as a result of numerous reactions during tea fermentation [3]. During the oxidation of polyphenolic compounds, catechins, flavonoids and other compounds with health-promoting properties emerge [4, 5]. The characteristics and composition of kombucha depend on various parameters, including the type of tea used, fermentation time, composition of the kombucha starter colony and fermentation temperature. In spite of the growing consumption of this beverage, our understanding of the effects of different fermentation parameters or the type of tea on the properties or chemical composition of the beverage is still incomplete [5].

Tea, and consequently kombucha, contains numerous minerals, including fluorine. Under physiological conditions, fluorine plays a significant role in hard tissue mineralisation processes [6–8]. However, there is little difference between the amount of fluorine which is beneficial for hard tissue mineralisation and a harmful dose. In addition, even at low concentrations, but with a long-term exposure, fluoride may accumulate in the body. The mechanism behind fluoride toxicity involves enhanced production of ROS (oxygen free radicals), increased lipid peroxidation and altered activity of many enzymes [6, 7, 9]. Additionally, fluorides easily cross cell membranes, entering soft tissues, which promotes their accumulation [6–8, 10]. Eighty to 85% of fluoride originates from food, in particular from drinks—tea, herbal infusions, coffee and alcoholic beverages, whereas the rest mainly comes from drinking water and toothpaste [11–17]. The concentration may be a result of multiple factors. Fluorine content in a product can be influenced by the origin of the resource and the age and type of the plant [13, 15, 18]. In the case of infusions or kombucha, other factors that should be considered include the method, the number of infusions made from the same resource, the time of brewing and the quality of water or tea used to prepare the infusions and fermentation process [13, 15, 18].

Therefore, the aim of this study was to evaluate the fluoride content in kombucha beverages made with the use of black, green, white and red tea at different time points of fermentation and ensure food safety.

## Material and Methods

### Plant Material

The material consisted of four types of leaf tea (*Camellia sinensis*): black Ceylon originating in India and green Gunpowder and white and red (Pu-ERH) originating in China or India.

### Preparation of Kombucha

The kombucha starter cultures, also known as SCOBY (which generally consists of *Acetobacter xylinum*, *Gluconobacter* spp., *Saccharomyces cerevisiae*), were obtained from a commercial source from Poland. The starter culture used in the present article was stored in a refrigerator (4 °C) and consisted of sour broth and cellulosic layer (SCOBY floating on the liquid surface). One hundred grams of sugar (100.0 g/L, 10.0%), 8 g of tea (8.0 g/L, 0.8%) and 1 L of hot, distilled water (90 °C) were mixed. The solution was infused for 10 min in a sterile conical flask. After cooling (30 °C), the tea decoction was filtered through nylon filters (0.45 μm, diam. 25 mm; Sigma-Aldrich, Poznań, Poland) into clean glass bottles and 10% of starter tea from a previous batch was added.

### Fermentation of Kombucha

Kombucha culture was kept under aseptic conditions. Fermentation was carried out by incubating the kombucha culture at 28 ± 1 °C for 7 and 14 days. Replicates were prepared and collected at each time point of fermentation process. The obtained kombucha was filtered and analysed.

### Determining Fluoride Content

F^−^ concentrations in individual samples were measured by the potentiometric method with a fluoride ionselective electrode (Orion 9409 BN, Thermo Scientific, USA). A total of 2.5 mL TISAB II and 2 mL trisodium citrate were added to the 0.5 mL sample. After mixing, the potential difference of each sample was measured for 10 min: 5 min before the addition of the appropriate F^−^ solution standard, and 5 min after the addition. According to the work of Łukomska et al., the F content in the samples was calculated based on the difference of potentials measured in each sample and the concentration of the added standard [15]. The electrode was calibrated using standard solutions. The correctness of the analytical procedure was controlled by comparing the concentration of F in the NaF solutions with the known concentrations: 0.1, 1.0, 10.0 mg/L (Orion Company, USA).

### The Determination of pH

The pH of both the fermented beverage and the unfermented control was determined using a pH meter (SCHOTT Instruments; SI Analytics Mainz, Germany).

### The Determination of Acetic Acid

Samples of tea and kombucha at 1, 7 and 14 days of fermentation were filtered through nylon filters (0.45 μm, diam. 25 mm; Sigma-Aldrich, Poznań, Poland). Acetic acid (AA) was analysed by high-performance liquid chromatography (HPLC) using a 1200 series HPLC connected to a 1100 series RI detector (Agilent Technologies, Santa Clara, CA, USA) with a Rezex ROA-Organic Acid H+ (8%) column (Phenomenex, Torrance, CA, USA). The column was eluted with a degassed mobile phase containing 5 mM H_2_SO_4_, pH 2.25 at 60 °C with a flow rate of 0.5 mL/min for 30 min per sample [19, 20]. The results are shown in mg acetic acid/L.

### The Determination of Alcohol

The ethanol content was measured using an alcoholometer (Browin, Łódź, Poland). The alcoholometer was immersed in the liquid and the result was read from the scale. Hydrometer was calibrated in alcohol by volume (alcoholic degrees).

### The Determination of Sugar Content

The total sugar content was measured with laboratory refractometer RL3 (Polish Optical Works, Warszawa, Poland) from Brix scale (g/100 mL).

### Statistical Analysis

In all the experiments, three samples were analysed and all the assays were carried out at least in triplicate. The statistical analysis was performed using Statistica 13.0 (TIBCO, Poland) and Microsoft Excel 2017. The results are expressed as mean values and standard deviation (SD). To assess the differences between examined parameters, the Tukey post hoc test was used. The Pearson test was used to calculate the correlation coefficient. Differences were considered significant at *p* ≤ 0.05.

## Result and Discussion

Fermented foods are increasingly popular, with consumers perceiving fermentation as a mild process for preserving food with major health benefits. Mild though it may be, fermentation and the processes it involves still have a significant impact on the quantity and quality of nutrients in the final product. The content of some nutrients, like sucrose, decreases, while others are found in higher quantities (acetic acid, alcohol). While tea is known as a significant dietary source of fluorine, there are few reports on the impact of fermentation on the concentrations of that mineral.

Our study shows for the first time that infusions made from kombucha prepared with different types of teas are a source of fluoride. However, it has to be considered whether the concentration of this element is low, or if the introduction of infusions to diets can pose a risk of excessive fluorine intake. Fluoride concentrations in the teas used in the preparation of fermented beverages ranged from 0.44 to 0.7 mg/L, and in kombucha from 0.42 to 0.93 mg/L. The highest fluoride content was found in green tea and the kombucha made from it, and the lowest in white tea. These differences may be related to the technology of preparing these teas. White tea is tea that has undergone little oxidation (or subsequent fermentation). During the long drying process, the cell structure of the tea leaves remains intact and the leaves are not damaged in any way by twisting or curling them. It is possible that the absence of damage may reduce the release of fluoride into the brew, but this requires further research in this area. In all samples, an increase in fluoride content was observed on day 14 of fermentation compared to the original tea. White tea was the only exception, with a relatively stable fluoride content in the tea and kombucha made from it (Table 1). Statistically significant differences are presented in Table 1. The recommended dietary allowance (RDA) in reference to fluorine is 3 mg/day [21], so one glass of kombucha would cover the need for the element in 7.75%. Importantly, the bioavailability of fluoride from liquids is much higher than that from solid foods, additionally increasing exposure to the mineral [22].

**Table 1:** Fluoride content in Kombucha tea

Fluoride content in kombucha [ppm]				
Day/tea	Green (GK)	Red (RK)	Black (BK)	White (WK)
0	0.70 ± 0.06*^c,d,g,j^	0.58 ± 0.00*^a,j^	0.54 ± 0.10*^a^	0.44 ± 0.06*^a,d,l^
7	0.66 ± 0.08*^c,e,g,k^	0.45 ± 0.03*^b,f^	0.46 ± 0.08*^b,i^	0.42 ± 0.08*^b,k^
14	0.93 ± 0.00*^a,b,f,i,l^	0.60 ± 0.14*^c,e^	0.67 ± 0.11*^c,h^	0.42 ± 0.04*^f,j,k,^

*p < 0.05 between type of kombucha (0, 7, 14 days of fermentation): ^a^GK 0, ^b^GK 7,^c^GK 14, ^d^RK 0, ^e^RK 7, ^f^RK 14, ^g^BK 0,^h^BK 7, ^i^BK 14, ^j^WK 0, ^k^WK 7, ^l^WK 14

Findings from our study regarding fluoride content in tea infusions are consistent with those of other authors. Buzalaf et al. [23] and Malinowska et al. [24] observed fluorine concentration at the range 1.21–3.56 mg/L and 0.57–3.53 mg/L, respectively. The study by Malinowska et al. [24] pointed to the following concentrations of fluoride: green tea 0.59–2.52 mg/L, green tea with additions 0.08–1.7 mg/L, oolong or Pu-ERH 0.39–2.85 mg/L, white tea 0.37–0.69 mg/L and herbal tea 0.02–0.14 mg/L. In turn, Gupta and Sandesh [25] determined fluoride content in black tea depending on the tea form: tea bags (1.67–2.67 mg/L), tea leaves (1.0–3.0 mg/L) and granulated tea (1.45–3.81 mg/L), and Maleki et al. [26] observed the level of this element in the range 0.75–3.29 mg/L in tea bags and 0.13–0.56 mg/L in leaves.

While numerous scientific reports confirm that tea is a major dietary source of fluoride, there has only been one study on the content of fluoride ions in kombucha [27]. However, the objective of that study was to develop a simple, fast and accurate method for determining the levels of ions, including fluoride ions, in tea infusions. The highest concentration in kombucha amounted to 3.2 mg/g [27]. There has also been a study measuring fluoride levels in traditional fermented alcoholic beverages (tella, tej, areki, shamita, borde and korefe). Fluoride concentrations ranged from 0.32 to 8.19 mg/L and depended on the type of beverage. As the potential contributing factors impacting on the variability in F^−^ content in the beverages, the authors mentioned differences in the topographical location of the raw material; differences in the mineral content of the soil, water and atmosphere; variation in the application of agrochemicals and variations in the beverage brewing process. A significant correlation was identified between the fluoride content in the water used in the preparation of the beverages and that in the final product. Therefore, both the quality of the source material and that of the water used in the preparation of the beverage affect the final outcome. It is worth adding that the present study used distilled water, while tap water used in a domestic environment and in the food industry will provide an additional source of fluoride.

Some authors have clearly proved that, the longer the tea brewing time, the higher the fluoride content in the tea [3, 24, 26]. Our findings also support the conclusion that longer fermentation is related to higher fluoride concentrations in the beverage. Still, on day 7, the content of fluoride ions was lower than that in the source material, i.e. the tea infusion. This may be due to several factors, including fermentation time and changes of the chemical parameters in kombucha, such as the overall content of sugar, alcohol, acetic acid and pH value. Moreover, the processes initiated by the SCOBY lead to the production of significant amounts of phenolic compounds. It has been suggested that some of those compounds are insoluble and may settle down at the bottom of the vessel as suspended particles, and due to their binding properties, they may adsorb fluoride ions, resulting in a lower fluoride content in the beverage [28].

Kombucha fermentation is a combination of three pathways of fermentation, i.e. alcoholic, lactic and acetic acid production. Lactic acid bacteria (LAB) are responsible for breaking down glucose. The yeast present in the kombucha culture converts glucose to ethanol, releasing carbon dioxide. In our study, alcohol content ranged from 0 to 3.5%. On fermentation day 7, alcohol level was the highest (3–3.5%). There is also a strong correlation between alcohol and acetic acid content for black tea in 7 days of fermentation (*r* = 0.9819; *p* < 0.05). On day 14, alcohol content decreased slightly in all the tested samples (2–3%) (Table 2). In a study by Gaggìa et al., alcohol content on day 14 was higher and amounted to 5.83% in black tea and as little as 1.14% in rooibos [19]. These differences may be associated with ambient temperature and type and amount of sugar, as well as the SCOBY community composition. No significant correlations were noted between fluoride content and alcohol concentration, which may be attributed to the narrow range of values. However, there are reports confirming the relationship between alcohol content and the parameters [14, 29]. Higher fluoride levels were observed in alcoholic beverages containing < 10% ethanol, like beer and wine, and lower in alcoholic beverages with > 40% ethanol content, as in vodka (0.044–0.131 mg/L) [29]. Those concentrations were nevertheless much lower than those found in our study, which may be due to the fact that kombucha was made from tea, which is a major source of fluoride itself. A low ethanol content (< 10%) may additionally contribute to such a high fluoride content.

**Table 2:** The content of alcohol, sugar, pH and acidity in kombucha tea

	Time points	Alcohol	pH	Sucrose	Acidity
	(day)	[%]		[° Brix, g/100 mL]	[mg acetic acid /100 mL]
Green tea kombucha – GK	Tea	0.00 ± 0.00*^3^	5.54 ± 0.01*^2,3,4^	10.75 ± 0.00*^2,3^	2.12 ± 0.01*^2,3^
	7	3.00 ± 0.00*^3^	2.61 ± 0.03*^1,5^	10.0 ± 0.00*^1,3,5,8^	703.92 ± 0.02*^1,5,8,11^
	14	2.75 ± 0.50*^1,2^	2.49 ± 0.04*^1^	8.75 ± 0.00*^1,2,3^	914.74 ± 0.31*^1,9,12^
Black tea kombucha – BK	Tea	0.00 ± 0.00	5.34 ± 0.03*^1,5,6,7,10^	11.0 ± 0.00*^5^	2.35 ± 0.01
	7	3.25 ± 0.50*^2,6,8,11^	2.62 ± 0.03*^2,4,6,11^	9.50 ± 0.00*^2,4,6^	703.91 ± 0.03*^2,8,11^
	14	2.00 ± 0.00*^4,5,9,12^	2.53 ± 0.03*^4,5,9, 12^	7.50 ± 0.00*^5^	908.30 ± 0.36*^4,5,9,12^
White tea kombucha – WK	Tea	0.00 ± 0.00	6.53 ± 0.05*^4,8,9^	10.75 ± 0.00	2.11 ± 0.01*^8,9^
	7	3.50 ± 0.50*^2,5^	2.53 ± 0.05*^7,9^	10.13 ± 0.00*^2^	704.81 ± 0.17*^2,5,7,9^
	14	3.00 ± 0.00*^3,6^	2.37 ± 0.05*^6,7,8^	9.50 ± 0.00	913.22 ± 0.43*^6,7,8,12^
Red tea kombucha – RK	Tea	0.00 ± 0.00	5.58 ± 0.07*^4,11,12^	10.75 ± 0.00*^11^	2.04 ± 0.03*^11,12^
	**7**	3.50 ± 0.50*^2,5^	2.38 ± 0.04*^5,10^	10.75 ± 0.00*^10,12^	705.95 ± 0.75*^5,10,12^
	14	3.00 ± 0.00*^3,6^	2.32 ± 0.02*^6,10^	9.50 ± 0.00*^11^	907.10 ± 0.62*^3,6,10,11^

*p < 0.05 between type of kombucha (7 and 14 days of fermentation) and tea; *p* < 0.05 between particular subgroup: ^1^GK 0, ^2^GK 7, ^3^GK 14, ^4^BK 0, ^5^BK 7, ^6^BK 14, ^7^WK 0, ^8^WK 7, ^9^WK 14, ^10^RK 0, ^11^RK 7, ^12^RK 14

The next step in the fermentation process involves the conversion of ethanol into acetic acid by bacteria of the genus *Acetobacter* [4, 5]. In this study, the pH value of the initial tea infusions was in the range 5.34–6.53. In kombucha, the parameter decreased significantly with fermentation time. No significant differences were observed in terms of pH value between beverages made from different types of tea (Table 2), which is consistent with the observations of other authors [30, 31]. The predominant acid found in kombucha is acetic acid, and its amount rises sharply in the course of fermentation [3, 5, 19, 31]. Acetic acid content was at its highest on day 14 of fermentation, amounting to respectively for green tea 914.74 mg/100 mL, white tea 913.22 mg/100 mL, red 907.10 mg/100 mL and black 908.30 mg/100 mL. These findings are consistent with other studies, where kombucha made with green and white tea was found to have the highest content of acetic acid [3, 5, 19, 31]. The amount of acetic acid increased with fermentation time, and consequently, the pH value decreased. This was accompanied by an increase in the amount of F^−^ in the analysed samples. Abu-Bakr et al. [32] also observed that low pH and increased acid content significantly affected the release of fluoride ions into beverages. In the present study, a positive correlation was found between fluoride content and acetic acid, irrespective of fermentation time for green tea (*r* = 0.7742, *p* < 0.05) and white tea (*r* = 0.8390, *p* < 0.05). Studies confirm that fluoride absorption into tea leaves is affected by pH [23, 33]. The highest F uptake was observed at pH 5.5 and at pH 4.0, it was significantly lower [33]. Moreover, the chemical equilibrium of F^−^ and HF and the availability of free fluoride ions depend on solution pH [33].

The sugar content in kombucha also changed over time and depended on the duration of the fermentation process. Refractometric determination of sugar content revealed the highest sucrose content in the initial tea infusions (11–10.75° Brix); with fermentation time, the amount decreased, reaching the lowest level on day 14 of fermentation (9.5–7.5° Brix). The initial increase in the content of reducing sugar may be attributed to the hydrolysis of sucrose to glucose and fructose by yeast. As fermentation progressed, sugar content dropped as it was used by yeast anaerobically to produce ethanol in the fermenting beverage [5, 19]. A strong correlation was observed between the decline in sugar content and fluoride concentration in kombucha, regardless of the day of fermentation for white tea (*R* = − 0.8007, *p* < 0.05), for red tea (*R* = − 0.5487, *p* < 0.05) and for green tea (*R* = − 0.7499, *p* < 0.05). However, there are no studies confirming this relationship in food.

## Conclusions

The F^−^ concentration in beverages is affected by the type of tea, sugar content—demonstrated for the first time in beverages—acetic acid and pH. For the sake of safety and preventing fluoride exposure, which can lead to many detrimental effects, including fluorosis, it is recommended to consume kombucha made with red and white tea in the first week of fermentation.
